# Mapping of QTLs and meta-QTLs for *Heterodera avenae* Woll. resistance in common wheat (*Triticum aestivum* L.)

**DOI:** 10.1186/s12870-023-04526-y

**Published:** 2023-10-31

**Authors:** Saksham Pundir, Rakhi Singh, Vikas Kumar Singh, Shiveta Sharma, Harindra Singh Balyan, Pushpendra Kumar Gupta, Shailendra Sharma

**Affiliations:** 1 Department of Genetics and Plant Breeding, Chaudhary Charan Singh University (CCSU), Meerut, Uttar Pradesh 250 004 India; 2 Department of Botany, Chaudhary Charan Singh University (CCSU), Meerut, Uttar Pradesh 250 004 India

**Keywords:** Wheat, Cereal cyst nematode (CCN), *Heterodera avenae*, Main effect QTLs, Epistatic interactions, meta-QTLs and candidate genes

## Abstract

**Background:**

In hexaploid wheat, quantitative trait loci (QTL) and meta-QTL (MQTL) analyses were conducted to identify genomic regions controlling resistance to cereal cyst nematode (CCN), *Heterodera avenae*. A mapping population comprising 149 RILs derived from the cross HUW 468 × C 306 was used for composite interval mapping (CIM) and inclusive composite interval mapping (ICIM).

**Results:**

Eight main effect QTLs on three chromosomes (1B, 2A and 3A) were identified using two repeat experiments. One of these QTLs was co-localized with a previously reported wheat gene *Cre5* for resistance to CCN. Seven important digenic epistatic interactions (PVE = 5% or more) were also identified, each involving one main effect QTL and another novel E-QTL. Using QTLs earlier reported in literature, two meta-QTLs were also identified, which were also used for identification of 57 candidate genes (CGs). Out of these, 29 CGs have high expression in roots and encoded the following proteins having a role in resistance to plant parasitic nematodes (PPNs): (i) NB-ARC,P-loop containing NTP hydrolase, (ii) Protein Kinase, (iii) serine-threonine/tyrosine-PK, (iv) protein with leucine-rich repeat, (v) virus X resistance protein-like, (vi) zinc finger protein, (vii) RING/FYVE/PHD-type, (viii) glycosyl transferase, family 8 (GT8), (ix) rubisco protein with small subunit domain, (x) protein with SANT/Myb domain and (xi) a protein with a homeobox.

**Conclusion:**

Identification and selection of resistance loci with additive and epistatic effect along with two MQTL and associated CGs, identified in the present study may prove useful for understanding the molecular basis of resistance against *H. avenae* in wheat and for marker-assisted selection (MAS) for breeding CCN resistant wheat cultivars.

**Supplementary Information:**

The online version contains supplementary material available at 10.1186/s12870-023-04526-y.

## Background

Common wheat (*Triticum aestivum* L.) is one of the important staple foods rich in protein and other micronutrients and is consumed as a major source for 20% of all calories and protein in human diet consumed by millions of people worldwide [1, 2]. The most destructive plant-parasitic nematodes (PPNs) like cereal cyst nematodes (CCNs) have an adverse effect on the production and associated grain quality traits in small grain cereals including wheat, particularly under drought and monoculture [3, 4]. In India, the disease *Molya* caused by CCN, *Heterodera avenae* is an important disease for oat (*Avena sativa*), barley (*Hordeum vulgare*) and wheat. *Molya* was first reported on wheat in India from *Neem Ka Thana* village in the Sikar district of Rajasthan state in 1958 (Vasudeva 1958, cited by Chhachhia and Kanwar [5]. The yield loss in wheat due to *H. avenae* ranged from 4.30 to 100% in different countries including Pakistan, India, Morocco, Saudi Arabia, Australia, United States, China, Tunisia, Syria, Turkey and Egypt [6–8].

*H. avenae* is one of the important sedentary and monocyclic species of CCNs complex that invades cereal crops only. The infective second-stage juveniles (J2s) of the nematode emerge from eggs, migrate into the soil, penetrate the root tips for their survival and multiplication and develop into the swollen white cyst (female) containing eggs, so that the roots of the infected plants become bushy, knotted and shallow [9, 10]. Although, chemical protection, rotations, different tillage systems and biological control are effective, but are not economical and practically viable options to prevent yield losses.

The use of wheat cultivars with resistance/tolerance is the most effective, economical and environmentally sustainable approach to prevent damage due to *H. avenae* [4, 11, 12]. Tolerant cultivars are characterized by their ability to withstand or recover from nematode invasion and give normal yield, because resistance suppresses or prevents the reproduction of the nematode, thereby reducing the density of inoculum available for invading the roots of the next cereal crop [13]. Plant resistance is also dependent on the genetic background and environment of the host and the pathogen with negative/positive effects on the expression of CCN resistance [14, 15].

Genetic studies on resistance to CCN have been conducted only sparingly. In one of the first genetic studies conducted in India, resistance was found to be monogenic and the genotype CCNRV 1 (Raj Molya Rodhak 1) was reported to be the only resistant wheat cultivar at that time [16]. Later, in another study, three resistant cultivars (Raj MR1, CCNRV 4 and AUS 15,854) were crossed with a susceptible cultivar (Raj 1482), demonstrating once again that difference between resistant and susceptible genotypes could be monogenic [17, 18]. However, there are studies, where resistance against CCN was shown to be quantitative in nature. As a result, 18 classical genes [19] and a number of QTLs were identified, which included the following: (i) three genes including *Cre1* (spring wheat, AUS90248 & AUS10894), *Cre8* (Festiguay) and *CreZ* (E-10 near isogenic line) in common wheat pool (ii) nine genes including the following: *Cre2, Cre5*, *Cre6 (Aegilops ventricosa*), *Cre3*, *Cre4* (*Ae. tasuchii*), *Cre7 (Ae. triuncialis*), *Cre9 (*Madsen), *CreX, CreY* (*Ae. peregrina*). These genes were obviously introgressed into wheat from the wild *Aegilops* species; (iii) one gene, *CreR* in *Secale cereal*; (iv) four genes (*Ha1*, *Ha2*, *Ha3* and *Ha4*) in barley; and (v) one gene (*CreV*) in *Dasypyrum villosum*. These genes exhibited complete or partial specific resistance to different CCN species/pathotypes reported from the different geographical regions [6–8, 11]. However, some of these resistance genes may provide resistance in only one region, while in other regions, popular cultivars with the same gene may prove to be susceptible, once again suggesting quantitative nature of nematode resistance, which is also controlled by the environment [8, 20, 21].

QTLs for CCN resistance have been largely identified using the two widely known methods, namely interval mapping [22–27] and genome-wide association analysis (GWAS) [28, 29]. Resistance against *H. avenae* has been reported in only a few hexaploid wheat cultivars from India, which suggest a need of further efforts to find novel genetic and genomic resources for resistance against *H. avenae* [30–34]. In the present study in wheat, interval mapping (including epistatic interaction analysis), and meta-QTL (MQTL) analysis were used to identify QTLs/MQTLs for resistance to *H. avenae*. Two repeat experiments were used to study the reproducibility of the current approach of recording resistance phenotype, since more than one approaches are available for recording data on nematode resistance. Candidate genes (CGs) underlying the MQTLs were also identified. The information on the genomic resources and the genes/QTLs collected during the present study may prove useful for understanding the molecular basis of resistance against *H. avenae* in wheat. The study may also help in marker-assisted selection (MAS), while breeding for nematode resistant wheat cultivars.

## Results

### Phenotypic variation

**Fig. 1:**
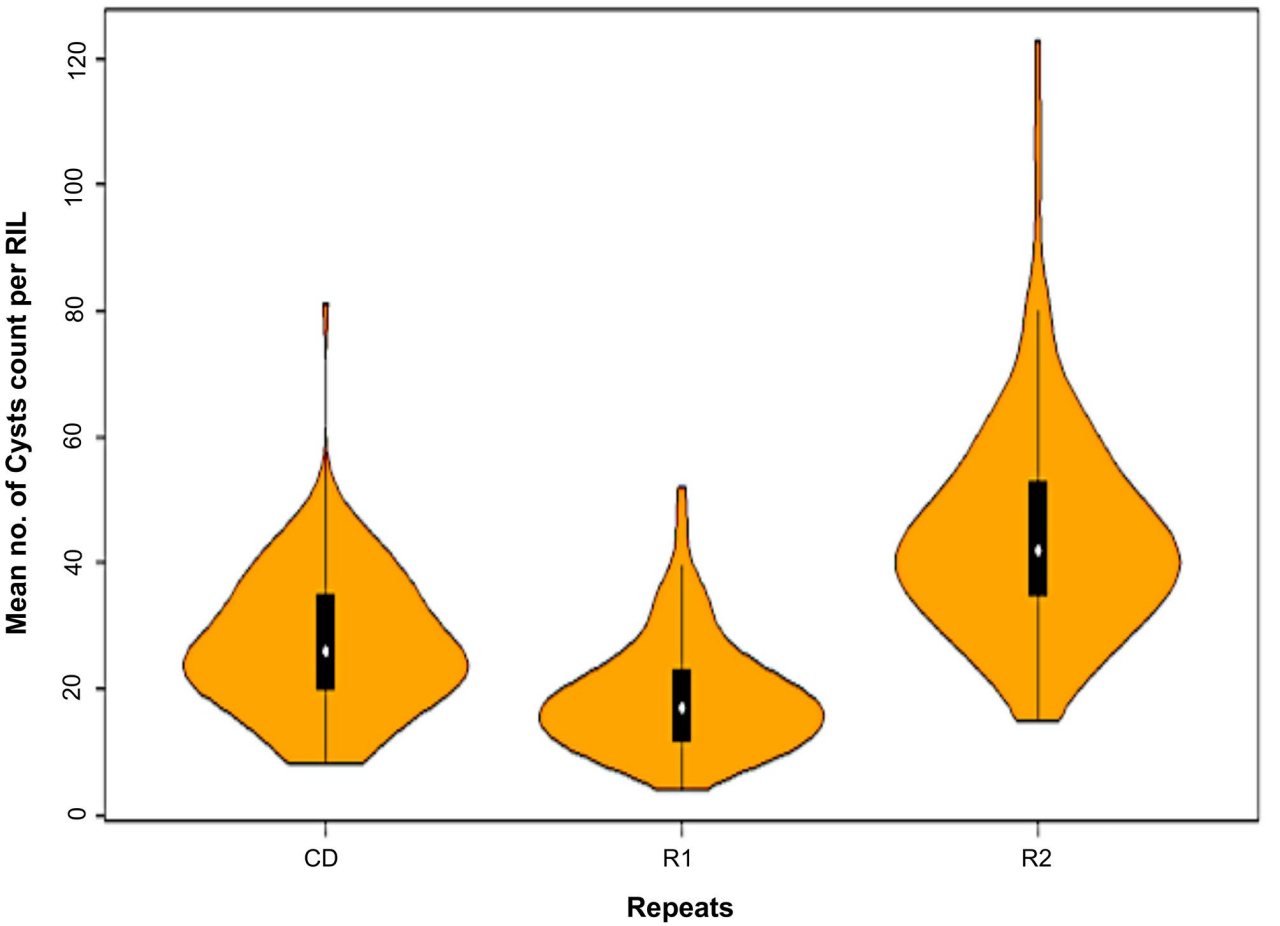
Violin plots showing the distribution of cysts count in RIL population. Shaded regions of the violin plots represent the frequency distribution of data. The vertical solid bar indicates the range of average values, and median is shown as a white circle, depicting the lower, medium and upper quartiles. Three sets of data [Repeat 1 (R1), Repeat 2 (R2) and combined repeat data (CD)] are shown on X-axis; cysts count are shown on Y-axis

The distribution of cyst count per plant on RILs is presented in Fig. 1, which shows a slightly positively skewed distribution in combined data analysis involving two repeats (R1 and R2). The range of cysts on individual RILs in R1, R2 and CD ranged from 1 to 123, and that of average number of cysts ranged from 18.66 to 43.44. The CV ranged from 1.70 to 2.36% across all repeats. The heritability estimates were 0.93, 0.57 and 0.76 for R1, R2 and CD, respectively. The Spearman’s rank correlation between the cysts on individual RILs in R1 and R2 was 0.32***. Variations due to genotypes, repeats and g × r interaction were highly significant, as revealed by ANOVA (Table 1).

**Table 1:** Details of the analysis of variance (ANOVA) for cysts count in RILs

Sources of variation	df	Mean Sq	F value
Repeats	1	11,504***	1454.767
Replication (Repeats)	6	1033***	13.062
Genotypes	148	427***	5.397
Genotypes × Repeat	148	300***	3.789

df: Degree of freedom; F: F distribution; Significant at: ‘***’ 0.001

### QTL analysis (CIM and ICIM analysis)

#### Main effect QTLs

Using CIM, six main effect QTLs were mapped on three chromosomes (1B, 2A and 3A) and using ICIM four main effect QTLs on each of the two chromosomes (1B and 2A) were detected; two QTLs, (*QCcnr.ccsu-2A.1* and *QCcnr.ccsu-2A.2*) were common, thus giving a total of eight QTLs (Table 2; Supplementary Fig. 1). These eight QTLs included three QTLs on 1B, four QTLs on 2A, and one QTL on 3A. The phenotypic variation explained (PVE) by individual QTLs ranged from 7 to 14%. The desirable allele contributing to resistance against *H. avenae* was contributed by cv. C 306 for all eight QTLs.

**Table 2:** QTLs for *H. avenae* resistance identified using composite interval mapping (CIM) and inclusive composite interval mapping (ICIM) in the RIL population

QTLs	Flanking Marker	Position (cM)	CI (cM)	Position (Kb)	LOD	PVE (%)	Add	R/CD
Composite Interval mapping (CIM)								
*QCcnr.ccsu-1B.1*	**WSNP4047**-WSNP3401	79.5	1.87	49,940,952	2.80	7	1.7926	R2
*QCcnr.ccsu-1B.2*	WSNP4047-**WSNP3401**	81.4	1.87	49,927,166	2.76	7	2.0095	CD
QCcnr.ccsu-2A.1*	**WSNP6862**-WSNP4934	18.0	22.81	31,045,433	3.62	14	2.8799	CD
***QCcnr.ccsu-2A.2***	WSNP6862-**WSNP4934**	22.8	22.81	50,038,871	2.59	7	2.2728	R1
*QCcnr.ccsu-2A.3*	**WSNP208**-WSNP5183	27.5	3.64	43,476,992	2.88	7	1.7865	R2
*QCcnr.ccsu-3A.1*	**WSNP1702**-WSNP4151	46.6	6.71	498,529,974	2.53	9	2.6697	R1
**Inclusive Interval Mapping (ICIM)**								
*QCcnr.ccsu-1B.3*	WSNP1974-**WSNP5998**	83.0	4	70,663,028	2.95	6	2.3018	CD
***QCcnr.ccsu-2A.1***	WSNP6862-**WSNP4934**	19.0	12	42,996,081	2.70	7	2.5686	CD
QCcnr.ccsu-2A.2*	WSNP6862-**WSNP4934**	22.0	9	43,263,254	3.37	8	2.3661	R1
QCcnr.ccsu-2A.4*	WSNP181-**WSNP6519**	210.0	13	747,233,694	3.35	7	2.1381	R2

QTL: Quantitative trait loci; cM: Centimorgan; kb: Kilobase; LOD: Logarithm of the odds; PVE: Phenotypic variability explained; %; Add: Additive effect; CI: Confidence Interval; R: Repeat; CD: Combined repeats data; R1: Repeat 1; R2: Repeat 2; QTL* = also detected in 1000 permutation test analysis

The marker closest to the QTL is indicated in bold. The two QTLs highlighted in bold were detected by both CIM and ICIM methods

#### Q × Q epistatic interactions

**Fig. 2:**
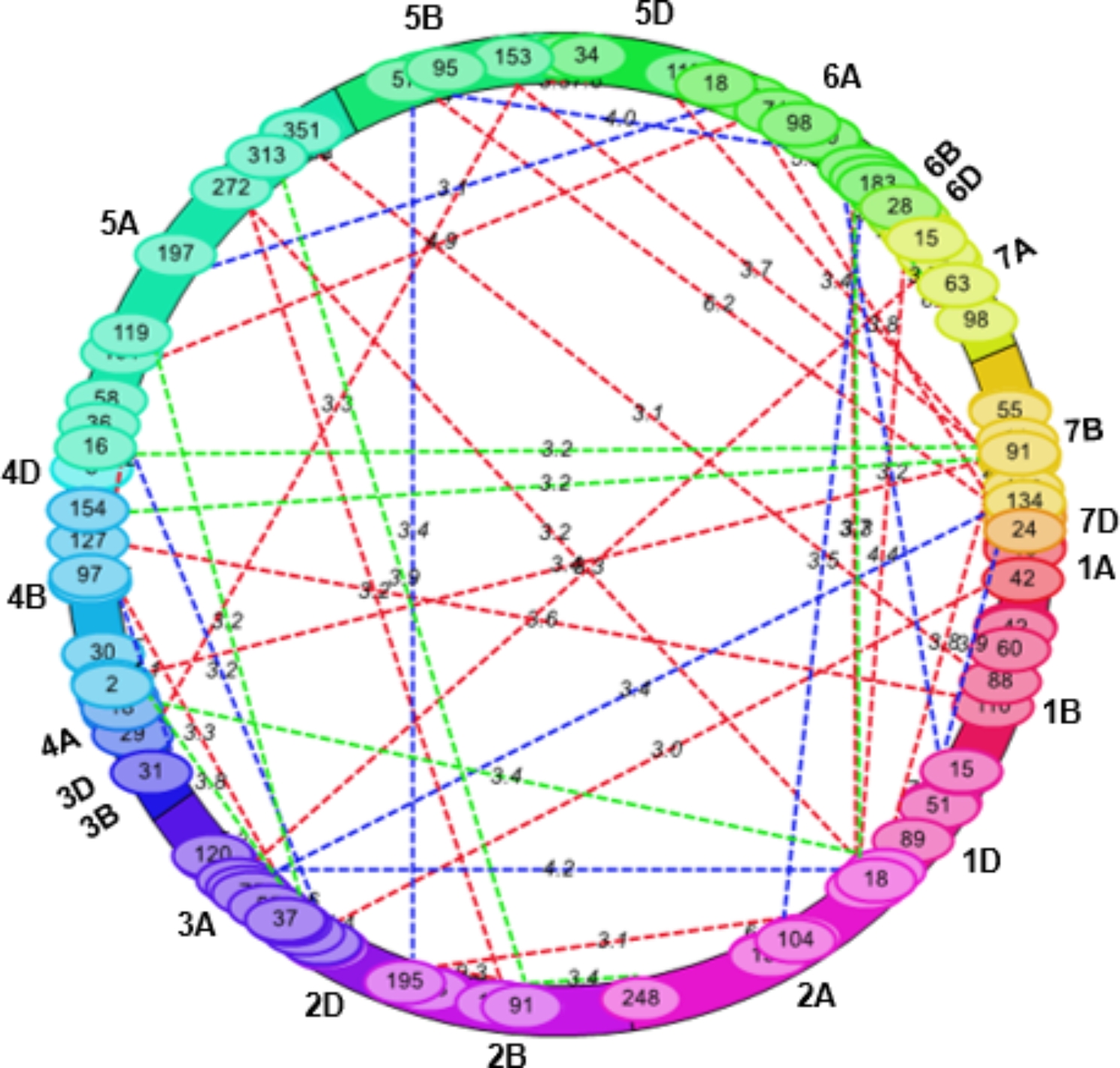
*H. avenae* resistance in common wheat; first order Q **×** Q epistatic interaction detected in (i) R1 denoted by red colour dotted lines, (ii) R2 denoted by blue colour dotted lines and (iii) CR data sets denoted by green colour dotted lines. Chromosome numbers (1A-7D) are shown outside the circle; numbers on dotted lines indicate LOD scores for individual epistatic interactions. The numbers on border of the circle are positions (in cM) of QTL 1 and QTL 2 involved in Q **×** Q epistatic interaction

Sixty-four (64) first-order epistatic (Q × Q) interactions were identified in two repeats and CD (Additional File 1: Table S1; Fig. 2), with a LOD score ranging from 3.05 to 9.33 and PVE ranging from 0.49 to 10.01%. One first-order interaction (*QCcnr.ccsu-5A.9* × *QCcnr.ccsu-5A.10*) was detected in both, R1 and R2. A summary of seven relatively more important Q × Q interactions is given in Table 3. Among two of the seven interactions, the additive × additive interaction effects were negative (*QCcnr.ccsu-2A.1* × *QCcnr.ccsu-6A.7* and *QCcnr.ccsu-2A.5* × *QCcnr.ccsu-4A.2*).

**Table 3:** Details of seven important Q1 **×** Q2 epistatic interactions for *H. avenae* resistance in the RIL population of common wheat

QTL1 (Flanking Markers)	QTL2 (Flanking Markers)	LOD	PVE (%)	Add×Add	R/CD
*QCcnr.ccsu-1B.5* (WSNP5306-WSNP2694)	*QCcnr.ccsu-1B.6* (WSNP5306-WSNP2694)	5.01	10.01	4.5409	R2
*QCcnr.ccsu-2A.1* (WSNP6862-WSNP4934)	*QCcnr.ccsu-6A.7* (WSNP6300-WSNP953)	3.27	7.49	-3.2833	CD
*QCcnr.ccsu-5A.9* (WSNP6033-WSNP2268)	*QCcnr.ccsu-5A.10* (WSNP2268-WSNP5502)	3.61	7.05	5.4322	R2
*QCcnr.ccsu-2A.5* (WSNP6862-WSNP4934)	*QCcnr.ccsu-4A.2* (WSNP6953-WSNP2905)	3.40	5.76	-3.1492	CD
*QCcnr.ccsu-3A.4* (WSNP5347-WSNP1228)	*QCcnr.ccsu-3A.5* (WSNP1228-WSNP6307)	3.62	5.38	7.5838	CD
*QCcnr.ccsu-7A.2* (WSNP5296-WSNP6353)	*QCcnr.ccsu-7A.5* (WSNP6143-WSNP5242)	3.74	5.35	3.9252	CD
*QCcnr.ccsu-7B.1* (WSNP6802-WSNP3017)	*QCcnr.ccsu-7B.2* (WSNP5949-WSNP3470)	3.81	5.35	6.2084	R2

R: Repeats: (R1: Repeat 1; R2: Repeat 2; CD: Combined repeat data); Chr: Chromosome; Pos: Position; LOD: Logarithm of the odds; PVE: Phenotypic variability explained; Add: Additive Effect

### Comparison with historical QTLs

**Fig. 3:**
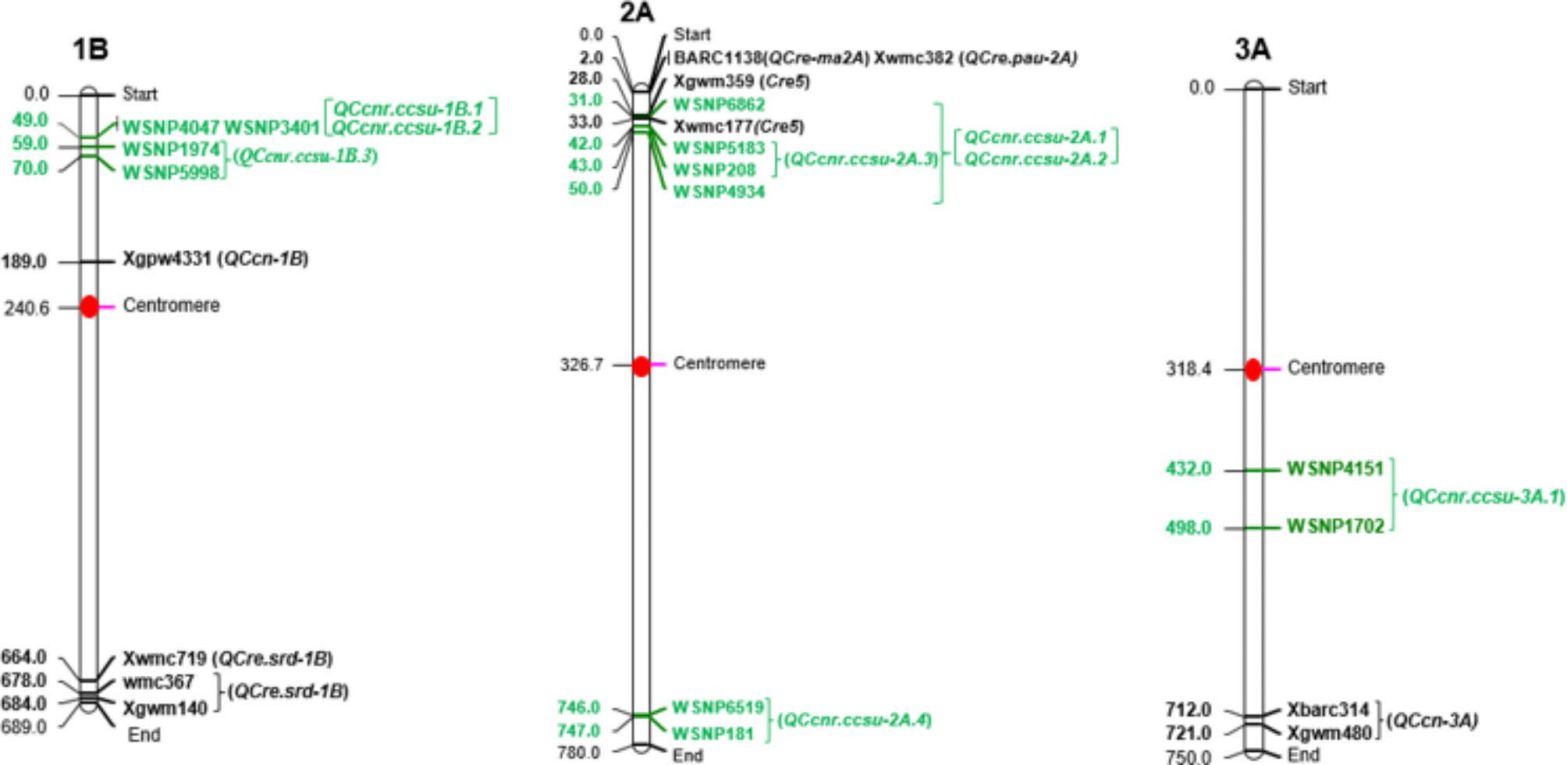
Distribution of QTLs/genes for *H. avenae* resistance on three chromosomes in common wheat reported in previous studies and the present study. The QTLs and associated markers detected during the present study are shown in green colour. Previously reported genes/QTLs are shown in black with associated/flanked markers (names of QTLs and associated markers are shown on the right and the distance (in Mb) between markers are shown on the left of each chromosome)

The physical positions of previously reported genes and QTLs are summarized in Tables S2 and S3 (Additional File 1). The representative chromosome maps showing QTLs detected in the present study along with previously reported QTLs or genes are shown in Fig. 3. When compared with known QTLs reported in earlier studies, the physical positions of three QTLs, namely *QCcnr.ccsu-2A.1*, *QCcnr.ccsu-2A.2* and *QCcnr.ccsu-2A.3* on chromosome 2A lie within or close to the physical position of the reported resistance gene, *Cre5*.

### Meta-QTL (MQTL) analysis

**Fig. 4:**
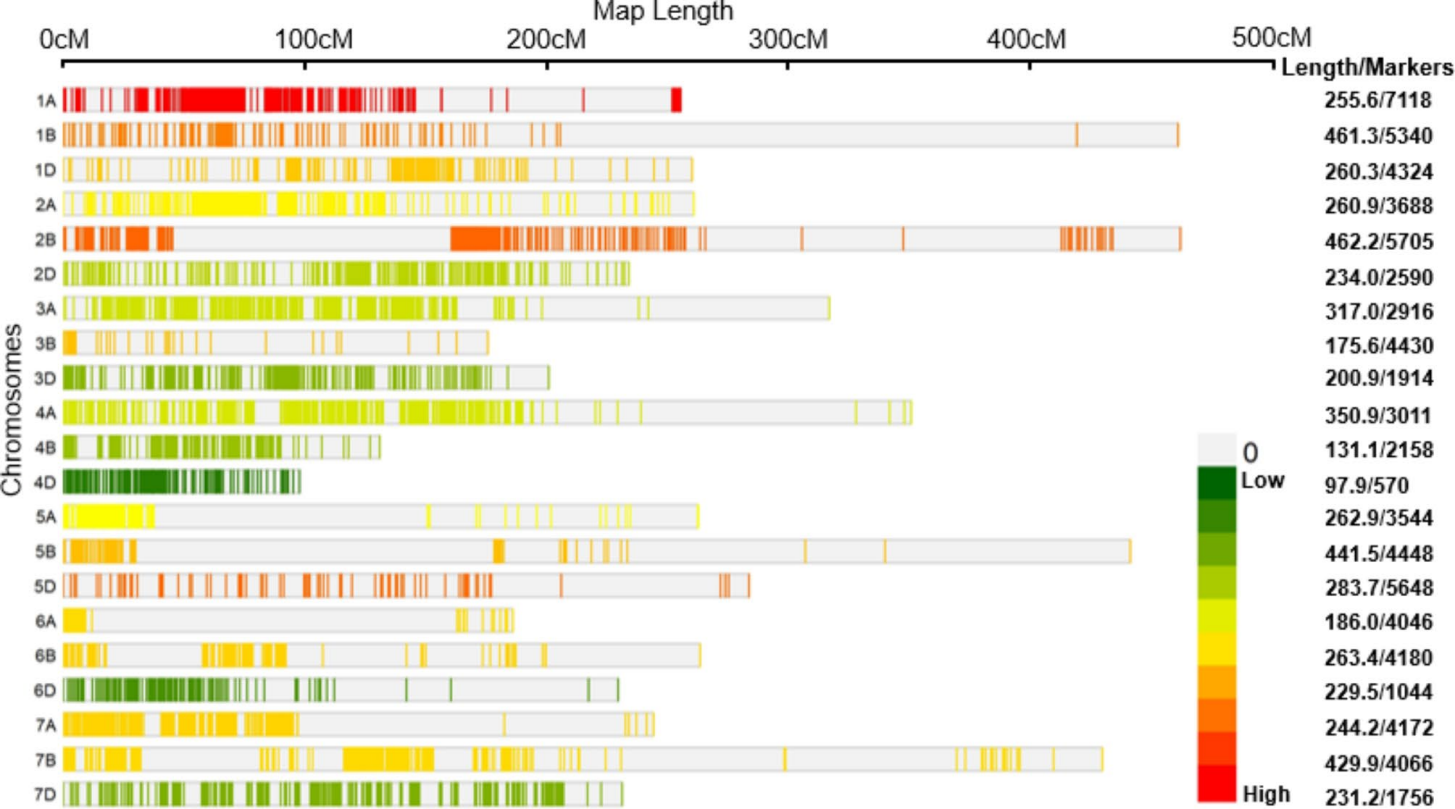
Marker densities (low to high) on each of 21 chromosomes of the consensus genetic map used for meta-QTL analysis. Green to red colors shows low to high densities of markers within each individual chromosome

The consensus genetic map for meta-QTL analysis, prepared during the present study had 76,668 markers (with an average of 3,651 markers per chromosome) with a genetic length of 5780.0 cM (Fig. 4). The marker types included the following: SNPs (77,137), SSRs (4,380), DArTs (3,526), and a variety of other markers (3,793) including AFLP, and STS markers. The maximum number of markers belonged to the B sub-genome (30,327) followed by A sub-genome (28,495) and D sub-genome (17,846). The size of the 21 individual linkage groups ranged from 97.9 cM (4D) to 462.2 cM (2B). The marker densities on individual chromosomes were relatively low and ranged from 0.04 markers per cM on 1A and 3B to 0.22 markers per cM for 6D.

Only 25 of the 34 known QTLs (PVE, > 0–70%; CIs, 0.80 to 22.81 cM) could be projected on the above consensus map, leading to the identification of only two MQTLs, (i) *MQTL1.2A* representing five QTLs; (ii) *MQTL2.2A* representing two QTLs (Table 4; Additional File 2: Supplementary Fig. 2). The remaining 18 original QTLs remained singletons.

**Table 4:** Meta-QTLs (MQTLs) for *H. avenae* resistance identified in common wheat

Meta-QTL	Flanking Marker	Chr	CI (cM)	Peak Pos (cM)	Peak Pos (Mb)	PVE (%)	No of QTLs
*MQTL1.2A*	BS00121553-BS00185908	2A	3.1	21.8	47.2	8.6	5
*MQTL2.2A*	gwm071d-BS00166296	2A	6.5	32.9	29.8	17.5	2

Chr: Chromosome; CI: Confidence Interval; Pos: Position; cM: Centimorgan; Mb: Megabase; PVE: Phenotypic variability explained

### Candidate gene analysis and *in-silico* gene expression

**Fig. 5:**
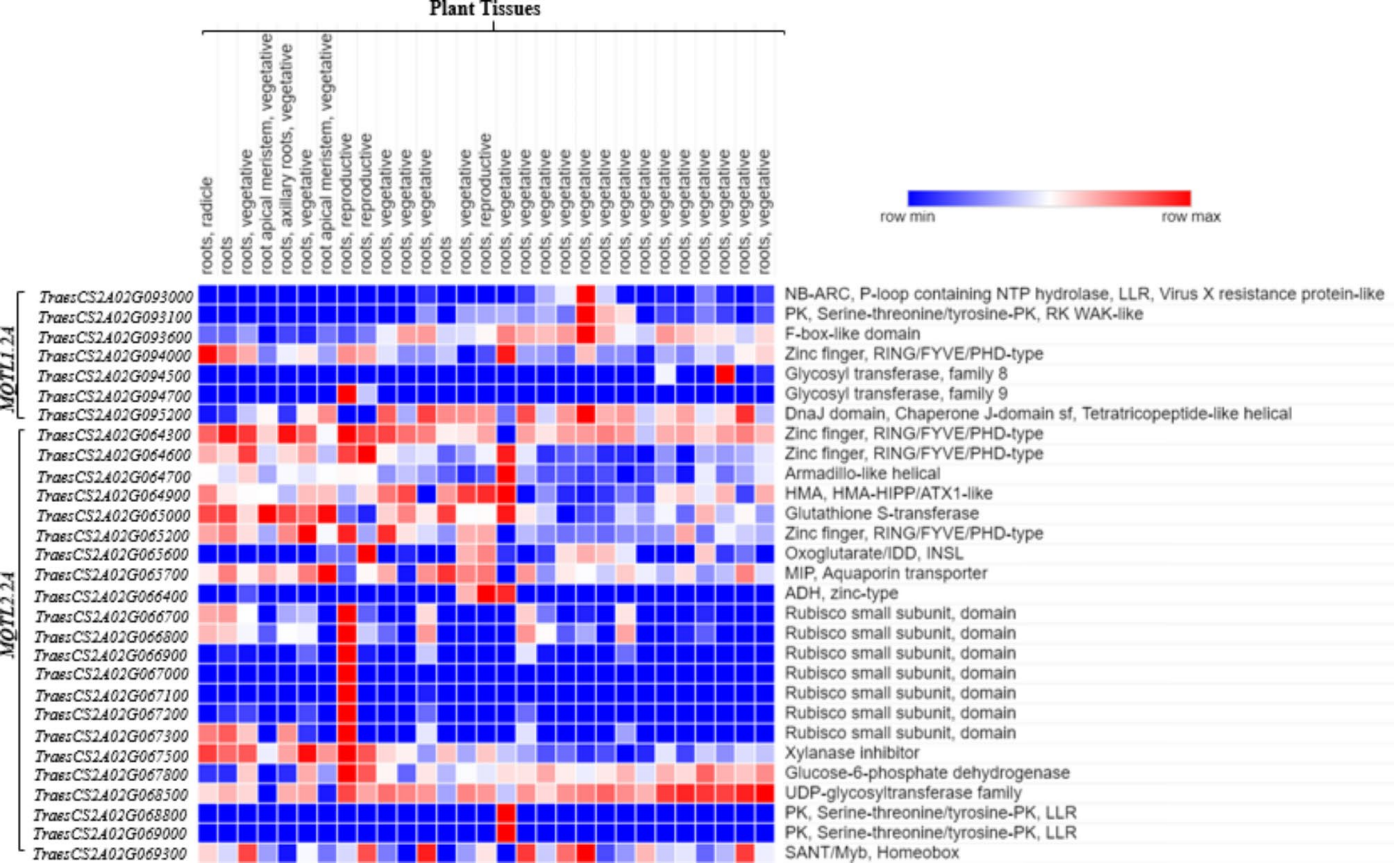
Expression profiles of candidate genes in wheat roots. Blue, white and red colours indicate low, medium, and high expression, respectively

Two MQTLs carried eighty-one (81) candidate genes (CGs); only 57 of these CGs apparently had a role in disease resistance (Table 5; for details see Additional File 1: Table S4). A majority of these genes showed differential expression in roots. We also selected 29 high-confidence CGs, with a majority showing an expression level of > 3 TPM (transcripts per-million) in wheat roots. The proteins encoded by these 29 CGs are listed in Table 5; Fig. 5 and Table S5.

**Table 5:** A list of important proteins encoded by 29 high-confidence candidate genes associated with two MQTLs for *H. avenae* resistance in wheat (protein domains in the encoded proteins by each CG shown in parentheses)

1. *MQTL1.2A*
(i) *TraesCS2A02G093000* (NB-ARC, P-loop containing nucleoside triphosphate hydrolase, Leucine-rich repeat domain; superfamily, Virus X resistance protein-like); (ii) *TraesCS2A02G093100* (Protein kinase-like domain superfamily, Serine/threonine-protein kinase, Receptor-like kinase WAK-like); (iii) *TraesCS2A02G093300* (Ubiquitin-like domain superfamily); (iv) *TraesCS2A02G093400* (Myb/SANT-like domain); (v) *TraesCS2A02G093500* and *TraesCS2A02G093600* (F-box associated domain, type); (vi) *TraesCS2A02G094000* and *TraesCS2A02G094800* (Zinc finger, RING/FYVE/PHD-type); (vii) *TraesCS2A02G094500* and *TraesCS2A02G094700* (Glycosyl transferase, family 8); (viii) *TraesCS2A02G094900* and *TraesCS2A02G095000* (Knottin, scorpion toxin-like superfamily); (ix) *TraesCS2A02G095200* (DnaJ domain, Chaperone J-domain superfamily)
**2.** ***MQTL2.2A***
(i) *TraesCS2A02G0638 # 200* and *TraesCS2A02G069300* (SANT/Myb domain, Homeobox-like domain superfamily); (ii) *TraesCS2A02G063900, TraesCS2A02G068600, TraesCS2A02G068700* and *TraesCS2A02G069100* (Protein kinase-like domain superfamily, Serine/threonine-protein kinase-like protein CCR3/CCR4); (iii) *TraesCS2A02G064300, TraesCS2A02G064600* and *TraesCS2A02G065200* (Zinc finger, RING/FYVE/PHD-type); (iv) *TraesCS2A02G064400* (Cytochrome P450, B-class); (v) *TraesCS2A02G064500* (Leucine-rich repeat, F-box protein, plant);(vi) *TraesCS2A02G064700* (Armadillo-like helical); (vii) *TraesCS2A02G064800* and *TraesCS2A02G064900* (Domain of unknown function DUF1618, Heavy metal-associated domain, Heavy metal binding protein HIPP/ATX1-like); (viii) *TraesCS2A02G065000* (Glutathione S-transferase); (ix) *TraesCS2A02G065300-TraesCS2A02G065600* and *TraesCS2A02G065800* (Oxoglutarate/iron-dependent dioxygenase, Isopenicillin N synthase-like); (x) *TraesCS2A02G065700* (Major intrinsic protein, Aquaporin transporter); (xi) *TraesCS2A02G065900* (Alpha/Beta hydrolase fold); (xii) *TraesCS2A02G066000* (F-box-like domain superfamily); (xiii) *TraesCS2A02G066400* and *TraesCS2A02G066500* (alcohol dehydrogenase, zinc-type); (xiv) *TraesCS2A02G066700-TraesCS2A02G067300* (Ribulose bisphosphate carboxylase small subunit, domain); (xv) *TraesCS2A02G067500* (xylanase inhibitor); (xvi) *TraesCS2A02G067800* (glucose-6-phosphate dehydrogenase); (xvii) *TraesCS2A02G067900, TraesCS2A02G068100-TraesCS2A02G068300* and *TraesCS2A02G068800-TraesCS2A02G069000* (protein kinase domain, serine-threonine/tyrosine-protein kinase, leucine-rich repeat = LRR); (xviii) *TraesCS2A02G068000* (leucine-rich repeat = LRR); (xix) *TraesCS2A02G068500*(UDP-glycosyltransferase family); (xx) *TraesCS2A02G069700* (papain C-terminal, papain-like cysteine endopeptidase superfamily)

## Discussion

The use of molecular markers for a study of genetic basis of disease resistance in general, and for a study of CCN resistance in particular, has been an important area of research during the last three decades [35]. These studies allowed identification of individual genes using Mendelian methods and identification of QTLs using QTL analysis based on DNA-based molecular markers. The molecular markers associated with these genes and QTLs can certainly be used for marker-assisted selection (MAS) for resistance breeding. Several studies have been conducted, where molecular markers associated with specific Mendelian genes were utlizized for breeding for nematode resistance; the crops where such attempts have been made include crops like soybean, peanut, cotton, tomato, cucumber, potato, turmeric, sugar beet, coffee, banana and wheat [36]. In wheat, SCAR markers associated with genes *CreX* and *CreY* have been used for resistance against *H.avenae* (a CCN used in the present study), and RAPD markers associated with *Mi* were used for resistance against *Meloidogyne incognita* (a root-knot nematode). But majority of these examples involve Mendelian genes and markers identified through QTL analysis have seldom been utilized for MAS. A solitary example of using a QTL involved pyramiding of a QTL for nematode resistance with another QTL for oleic acid content in peanut [37].

QTL analysis is often followed by several approaches including the following: (i) meta-QTL analysis can be conducted to obtain more robust markers; (ii) fine mapping may be undertaken for identification of closely linked markers or for cloning of genes underlying the QTLs for resistance [38]. (iii) Candidate genes associated with QTLs or metaQTLs may be identified using *in silico* approaches. In the present study, we carried out QTL interval mapping, meta-QTL analysis and identification of candidate genes for resistance against *H. avenae* in common wheat. The results obtained in this study are discussed using the findings of earlier studies on the subject.

### Reproducibility in two repeats

The number of cysts recorded on the individual RILs in the two repeats differed; the Spearman’s rank correlation between cyst count of the nematode/RIL in the two repeats was relatively low, but significant (r_s_ = 0.32***). This suggests that the reproducibility of data on resistance is satisfactory and that the patterns do not differ widely. A significant genotypes × repeat (g×r) interaction observed in ANOVA during the present study may be attributed to differences in microenvironments. However, the ranks of RILs and those for number of cysts in R1 and R2 are similar. This suggests that the level of resistance in different RILs inferred in R1 and R2 do not differ, which supports the conclusion that the differences are due to microenvironment. Similar results were also reported in the previous studies on CCN resistance in wheat from our own laboratory [39, 40]. The occurrence of g×r interaction has perhaps interfered with detection of some QTLs for CCN resistance in both the repeats, so that none of the eight QTLs detected in the current study was discovered in both the repeats (R1 and R2), although some QTLs were detected in the combined data (CD).

### Main effect QTLs and Q × Q epistatic interactions

The results of the present study suggest that QTL analysis should be conducted using both CIM and ICIM, because the results differ, suggesting that more QTL can be identified using both the approaches. Also, the two QTLs (*QCcnr.ccsu-2A.1* and *QCcnr.ccsu-2A.2)* detected using both approaches should be considered to be important, and one can use such QTLs with higher level of confidence. The QTLs detected by only one approach, including four QTLs detected by only CIM (*QCcnr.ccsu-1B.1* and *QCcnr.ccsu-1B.2*, *QCcnr.ccsu-2A.3, QCcnr.ccsu-3A.1*) and two QTLs detected by only ICIM (*QCcnr.ccsu-1B.3, QCcnr.ccsu-2A.4*) deserve careful further scrutiny. We believe that the two different methods, namely CIM and ICIM are complementary, although the developers of ICIM (Dr Jiankang Wang and his group in China and CIMMYT) claim that ICIM is an improvement over CIM [41]. In the present study, the eight main effect QTLs for *H. avenae* resistance were detected on three chromosomes (1B, 2A, and 3A). These results differ from the results reported in previous studies where QTLs for resistance to *H. avenae* resistance in wheat were reported on a total of 13 different chromosomes, although these 13 chromosomes included the three chromosomes identified during the present study [22–27]. Further GWAS conducted for CCN resistance in wheat also reported MTAs on 14 chromosomes including the three chromosomes, which carried the QTLs reported in the present study; for details of MTAs, see reference [39].

The eight QTLs identified by CIM and ICIM in the present study, when compared with QTLs reported in earlier studies, suggest that majority of the QTL identified in the present study are novel and were not identified in earlier studies [22–26]. However, the physical positions of at least some of these eight QTLs (particularly the one on chromosome 1B) reported during the present study are different from those reported earlier (see Fig. 3). The identification of novel QTLs in the present study may be attributed to the use of different mapping populations derived from parental genotypes, which differ from those involved in mapping populations used in earlier studies. It has been repeatedly emphasized that one of the weaknesses of QTL interval mapping relative to genome-wide association studies (GWAS) is the sampling of limited genetic material involving only two parents, such that additional interval mapping with new parents, is always desirable. The present study represents one such effort.

**Fig. 6:**
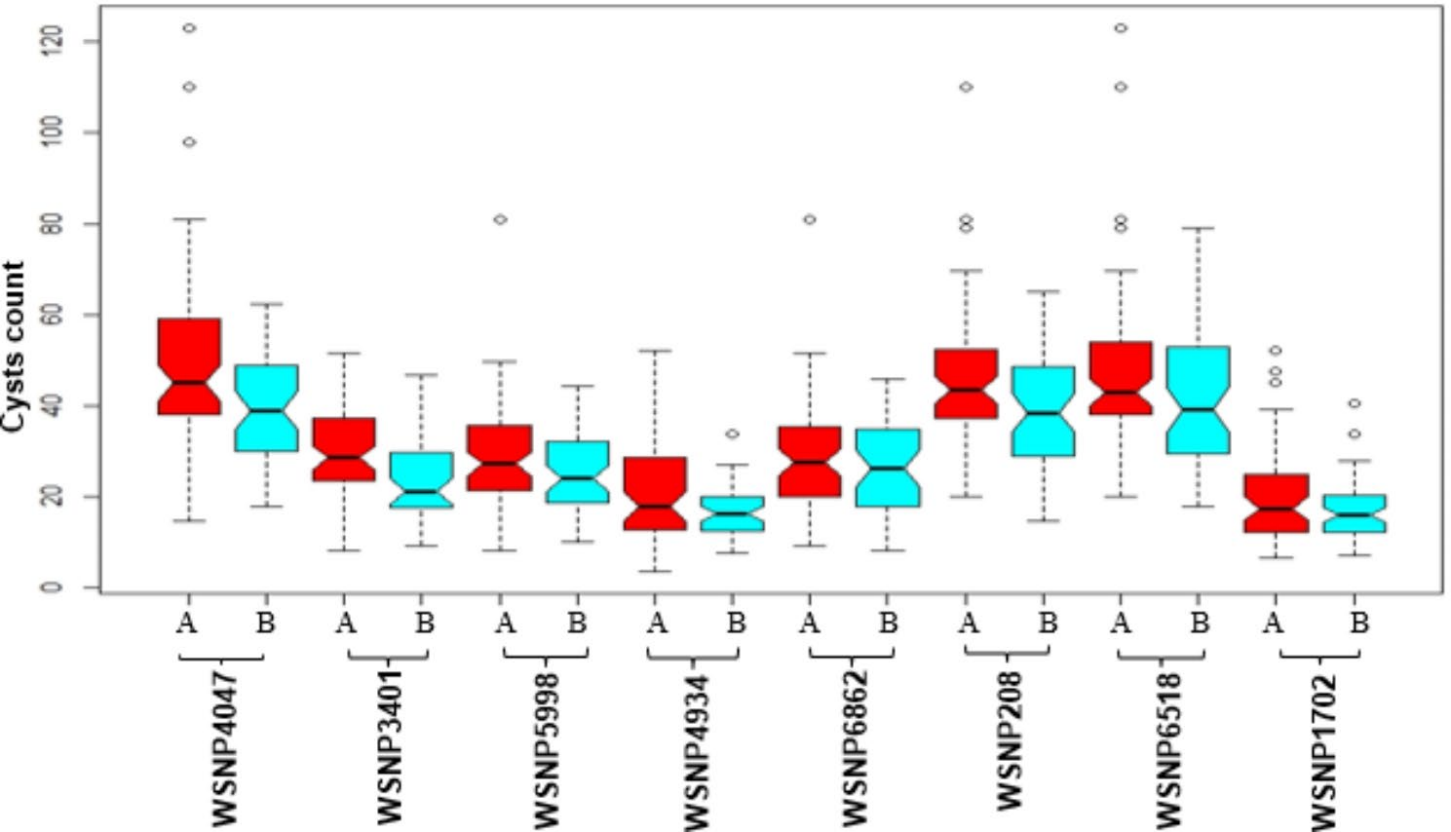
A box plot showing the effect of allele of closest marker to QTLs on 1B, 2A and 3A contributing for *H. avenae* resistance. Middle vertical lines show the medians; box limits indicate the 25^th^ and 75^th^ percentiles; whiskers extend to 5^th^ and 95^th^ percentiles, outliers are represented by dots. Markers (with marker names and alleles) are shown on X-axis; cyst counts are shown on Y-axis. ‘A’ allele belongs to the susceptible cv. HUW 468 and ‘B’ allele belongs to the moderately resistant cv. C 306

Interestingly three QTLs (*QCcnr.ccsu-2A.1*, *QCcnr.ccsu-2A.2* and *QCcnr.ccsu-2A.3*) identified during the present study and QTLs reported in earlier studies (all located on short arm of 2A) lie in the vicinity of the *Cre5* gene for resistance to *H. avenae* [22–25]. The location of the *Cre5* was detected within the interval flanked by the markers associated with the above QTL region on chromosome segment (2NS) introgressed from *Ae. ventricosa* [42]. Several common wheat cultivars containing a segment from chromosome 2NS of *Ae. ventricosa* in the chromosome arm 2AS were also reported to be resistant to European and Australian pathotypes of *H. avenae* [43, 44]. Of the three QTLs located in the region carrying *Cre5* gene, the QTL *QCcnr.ccsu-2A.1* also had the highest PVE of 14% and, therefore these three QTLs may be important for resistance against *H. avenae* in common wheat. The remaining five QTLs with PVE ranging from 6 to 9% constitute additional important genomic regions, which may also be exploited along with other sources of CCN resistance in breeding for resistance against *H. avenae* in common wheat using recurrent selection. The desirable alleles for all eight QTLs were contributed by the tall parent cv. C 306 (Fig. 6), and therefore, user-friendly KASP markers may be designed based on the SNP alleles of the markers flanking the QTLs that are present in the parent cv. C 306 for improvement of resistance to *H. avenae* in common wheat through marker assited recurrent selection (MAS), since desirable alleles will be scattered in different plants in the segregating population.

Another important aspect of the present study is the identification of seven important first-order additive × additive interactions that altogether involved three main effect QTL (*QCcnr.ccsu-2A.1*, *QCcnr.ccsu-2A.2* and *QCcnr.ccsu-2A.3*) and six E-QTLs (epistatic QTLs) (Table 3; SI 1). This suggested that there may be QTLs that do not have their own main effect but are involved in epistatic interactions only. Some previous studies in common wheat have also reported E-QTLs along with main effect QTLs for yield-related traits including plant height, spike shattering and flour colour, salt tolerance, stripe rust and leaf rust resistance [45–53]. These seven first-order interactions have PVE ranging from 5.35% to 10. 01% and together make 46.39% PVE. Two of these seven first-order interactions have negative additive × additive interaction effects (the remaining five interactions have a positive effect) and thus are desirable for improving resistance against *H. avenae* in breeding. The utility of such additive × additive interactions has been suggested in wheat for improvement of NUE (Nitrogen Use Efficiency) and other traits in several previous studies in wheat [54–57].

### Meta-QTLs (MQTLs)

The present study is also the first study on MQTL analysis for resistance against *H. avenae* in common wheat. Two MQTLs for *H. avenae* resistance, both located on chromosome 2AS were identified during the present study (Table 4; Supplementary Fig. 2). These two MQTLs were closely placed in a QTL hot spot region that has been repeatedly shown to harbour QTLs and *Cre5* gene for resistance to *H. avena*e in common wheat. The confidence interval (CI) of the MQTLs is much smaller (mean = 4.86 cM; range = 3.14–6.59 cM) than the CI of the original QTLs, where it ranged from 0.8 cM to 22.8 cM with a mean of 7.69 cM, suggesting the more precise mapping and robust nature of the MQTLs. Importantly, the PVE% due to the MQTLs was also higher than the main effect QTLs and ranged from 8.6 to 17.5% suggesting that these MQTLs may be important candidates for breeding for resistance to *H. avena*e. The occurrence of only two MQTLs for *H. avenae* resistance during the present study is not surprising because as few as one to two MQTLs have also been reported for traits like grain yield, flowering time/photoperiod sensitivity in the previous studies in wheat [58, 59], although, much larger number of MQTLs have also been reported for some other traits in common and durum wheat [60–62]; for more details see reference [63].

### Candidate genes

Out of 81 candidate genes (CGs) identified during the present study, protein products of 57 genes were known; 29 of these CGs had high expression in roots (Additional File 1: Table S4) and encode proteins that are involved in providing resistance against different types of pathogens in a variety of plant systems (Table 5). This suggests their possible potential role in nematode resistance. One of the identified gene *TraesCS2A02G093100* associated with *MQTL1.2A *encode for Receptor-like kinase WAK-like protein. Notably, cloning of the QTL *QFhb.mgb-2A* led to the identification of wall-associated receptor-like kinase (WAK2) gene responsible to Fusarium Head Blight resistant in wheat [64] thus suggesting that this gene provide resistance to both the fungal and CCN pathogens in wheat. However, further functional characterization of the other genes identified during the present study may help in the identification of genes involved in CCN resistance in wheat.

## Conclusions

Based on the results of the present study, we propose that the two MQTLs on chromosome 2A that are considered to be more precise and robust with narrow CI along with two additive × additive interactions (*QCcnr.ccsu-2A.1* × *QCcnr.ccsu-6A.7* and *QCcnr.ccsu-2A.5* × *QCcnr.ccsu-4A.2*) that have negative additive effect may be exploited for breeding for CCN tolerance/resistance in wheat. For this purpose, user-friendly KASP markers for the two MQTLs (on 2A) and the QTLs involved in the above two first-order interactions may be developed and used in the marker-assisted recurrent selection (MARS) in segregating breeding populations for breeding CCN tolerant/resistant wheat genotypes. Also, after due functional characterization of the CGs involved in CCN resistance identified during the present study, suitable gene-based functional markers may be developed for use in MAS in future breeding programmes aimed at breeding for CCN resistance in wheat.

## Methods

### Plant material

A mapping population comprising 149 F_9_ recombinant inbred lines (RILs) derived from the cross HUW 468 × C 306 was used. Seed material of the population was kindly provided by Professors AK Joshi and VK Mishra of BHU, Varanasi. The female parent of the cross (HUW 468) is a dwarf cultivar that was developed by BHU, Varanasi and the male parent (C 306) is an old tall Indian wheat cultivar that was developed at CCSHAU, Hisar, Haryana. These two genotypes are diverse with different pedigrees, as follows: (i) HUW 468: CPAN-1962 / TONI //LIRA’S’/ PRL’S’, and (ii) C 306: RGN/CSK3//2*C5 91/3/C217/N14 //C281.

### Screening of parental genotypes and RIL mapping population

Initially, 10 plants each of the two parental genotypes were screened, in batches, against the prevalent local *H. avenae* strain from North India. Parent C 306 was moderately resistant and HUW 468 was susceptible. Subsequently, all RILs, the two parental genotypes, one resistant check, namely wheat cv. RAJ MR1 [33] and two susceptible checks, namely WH 147 and PBW 343 [31, 65] were screened for resistance against *H. avenae* population.

### CCN inoculum and phenotyping

Cysts of CCN were extracted from the heavily infested soil from the Hissar district of Haryana, India by decanting and sieving methods [66]. Cysts were surface sterilized with 0.5% sodium hypochlorite (NaOCl or bleach) for 10 min and rinsed six times with distilled water. The cysts obtained thus were stored at 4 °C for two months. The juveniles at J2 (second juvenile stage) were transferred to room temperature (~ 25 °C) for 24 h; these were then used for inoculation following Fisher [67].

The experiment was conducted in a completely randomized design (CRD) under controlled conditions (22 °C ± 2, 16 h light, 8 h darkness and ~ 65% relative humidity) within two batches. In batch 1 (repeat 1) and batch 2 (repeat 2), five and three replications of each line were grown, respectively. Seeds were pre-germinated on a wet filter paper and then transferred into 150 cm³ PVC (polyvinyl chloride) pipes, each filled with soil that was sieved and double steam-sterilized. After 10 days of plant emergence, each pipe was inoculated by pipetting with 1ml inoculum/plant containing 1000 nematodes (J2s) in three holes around the base of the stem and the roots; after inoculation, these were covered with soil. The plants were also irrigated with Hoagland media at a fixed interval to maintain their growth and health.

White and brown cysts were extracted from both soil and plant roots after 75 days of inoculation and counted for each plant under a stereomicroscope (Nikon SMZ645). Roots and sieved soil from roots were later also examined for the absence of cysts, and to confirm that all cysts were used for counting [27]. The genotypes were placed in the following five groups based on the mean number of cysts per plant: (i) resistant (R) with fewer cysts than in the known resistant check; (ii) moderately resistant (MR) with the number of cysts, little higher than in the resistant check; (iii) moderately susceptible (MS) with more cysts than in the resistant check, but not as many as in the susceptible check; (iv) susceptible (S) with as many cysts as in the susceptible check and (v) highly susceptible (HS) with many more cysts than in the susceptible check [28, 68].

### Statistical analysis

Statistical analysis of 149 RILs was done for each of the three data sets: (i) Repeat 1 (R1), (ii) Repeat 2 (R2) and (iii) combined repeats data (CD). Analysis of variance (ANOVA) of cysts count obtained through phenotyping was conducted in RStudio (R Core Team 2016).

Best linear unbiased prediction (BLUP) values were computed for utilization in QTL analysis. The employment of random effects models was carried out through the utilization of the R package lme4 within the MetaR software [69]. Multi-environmental models were exclusively established incorporating random effects such as replication (R) within the repeats (r), genotype (g), genotype x repeats and repeats.

$${Y = b + \beta_{1}R(r)+\beta_{2}g+\beta_{3}gr+\beta_{4}r+\varepsilon}$$

Where, Y represent the array of phenotypes. The symbol b stands for the intercept, while *β1*, *β2*, *β3*, and *β4* are the coefficients associated with the independent variables. Lastly, ε represents the presence of stochastic error.

The broad-sense heritability (H^2^b) for resistance to cereal cyst nematode (CCN), *Heterodera avenae* was calculated using the equation as:

$${H^{2}b={Vg/Vp}}$$

Where Vg (Vg = [MSG - MSE]/R) is genotypic variance and Vp (Vp = Vg + Vr) is phenotypic variance which was calculated as suggested by Comstock and Robinson [70]. The Spearman’s rank correlation coefficient (r_s_) between the cysts count of the RILs in the two repeats was also calculated using standard methods.

### Linkage map

A genetic map based on 451 SNP loci covering a genetic distance of 2558.52 cM distributed on on 21 chromosomes, developed in our recent study [57] was used for QTL interval mapping. The genotyping data was based on genotyping-by-sequencing (GBS).

### QTL interval mapping and Q × Q epistatic interaction analysis

Composite interval mapping (CIM) and inclusive composite interval mapping (ICIM) were, respectively, conducted using QTL Cartographer V.2.5 [71] and IciMapping v4.2 [72] to identify QTLs. QTL analysis was separately conducted using three data sets, namely R1, R2 and CD. In CIM, likelihood-of-odds (LOD) thresholds were calculated by two methods, (i) LOD ≥ 2.5 and (ii) 1000 permutations at *p*-value ≤ 0.05 LOD significance thresholds. In ICIM also, LOD thresholds were calculated by two methods, (i) LOD ≥ 2.5 and 1000 permutations at *p*-value ≤ 0.01 LOD significance thresholds, scanning step = 1.0 cM using ICIM of the additive (ICIM-ADD) tool. Inclusive composite interval mapping of Q**×**Q epistatic QTL (ICIM-EPI) functionality was also used at *p*-value ≤ 0.01, LOD = ≥ 3.0 significance thresholds, scanning step = 1.0 cM to detect possible Q**×**Q epistatic interactions between QTLs.

### Meta-QTL (MQTL) analysis

#### Retrieval of data on QTLs

For conducting MQTL analysis, information on known QTLs for resistance to *H. avenea* from all the six studies published till 2022 on interval mapping was retrieved as mentioned in SI6. Following two types of input data text files were prepared from each study following Biomercator v4.2 [73, 74]: (i) genetic map file, and (ii) QTL information [name of QTL, trait, chromosomes carrying the QTLs, range and mean of the lengths of confidence interval (CI), LOD score, R^2^ etc.]. If the value of CI for a particular QTL was not available in the original study, it was worked out using following formulae: (i) for F_2_ and BC: 530/N.R^2^ [75]; (ii) for RILs: CI = 163/(N.R²) [76]; and (iii) for DH: CI = 287/(N.R²) [77], where in each case, N is size of the mapping population and R^2^ is the phenotypic variation explained (PVE).

#### Development of consensus map

Consensus genetic map of wheat was developed using LPmerge software [78] using six published linkage maps [79–84]. Markers flanking individual QTLs were also included in the consensus genetic map. The consensus map was generated using R-based ‘LPMerge’ package [78]. The length of the individual chromosomes was calculated following Hubert and Hedgecock [85].

#### Projection of QTLs on consensus map and identification of MQTLs

The consensus map was used for the projection of QTLs reported in six earlier studies following BioMercator v 4.2 manual [73, 74]. The QTLs that did not had information for PVE value, LOD score or genetic position, etc. were not used for projection. Based upon the available number of QTLs, we used the method proposed by Goffinet and Gerber [86] for the projection of QTLs. In this case, the number of underlying MQTLs that best fit the available results is determined following testing all possible combinations based on the Akaike Information Criterion (AIC). The QTLs which could not be projected onto the consensus map or those with mapping positions outside the consensus map were excluded.

### Identification of candidate genes and *in-silico* gene expression

For the identification of putative candidate genes (CGs) and their gene ontology, the following steps were used: (i) Nucleotide sequences of the flanking markers for specific MQTL were retrieved either from CerealsDB or from IWGSC database; these sequences were utilized to identify the physical positions employing nucleotide blast (maximum E-value = 1E-100 and minimum 95% identity of the sequence) against wheat reference genome sequence available in EnsemblPlants (*Triticum_aestivum* IWGSC_ensembl_release 48/IWGSC RefSeq v1.1). (ii) The physical interval (in Mb) for an individual MQTL was calculated using the genetic confidence interval (in cM) of the MQTL regions. For this purpose, the physical interval (in Mb, calculated from the coordinate information of the MQTL) was divided by the genetic interval (in cM) and the distance in units of bases per cM was calculated. (iii) Actual physical position of the MQTL was calculated and 1 Mb region on either side of the MQTL peak (total 2 Mb intervals) was used for identification of the CGs associated with MQTL region. (iv) Annotation of CGs was undertaken on the basis of the domain in the corresponding protein sequences, which were obtained using InterPro database.

The CGs were subjected to *in-silico* expression analysis considering different tissues and developmental stages of wheat via the ‘Wheat Expression Browser-expVIP’ (expression Visualization and Integration Platform; http://www.wheat-expression.com) [87, 88]. Among all tissues, roots were selected to check the expression of CGs at specific developmental stages.

### Comparison of QTLs detected to historical QTLs

The QTLs identified in the present study were also compared with previously reported QTLs for resistance to *H. avenae.* For this purpose, the physical positions of all QTLs detected in the present study and those reported previously were located through Ensembl Plants (IWGSC RefSeq v1.1). The positions of QTLs detected in the present study and previously reported QTLs on common wheat chromosomes were located using Map chart software.

### Electronic supplementary material

Below is the link to the electronic supplementary material.

Supplementary Material 1

Supplementary Material 2

## Data Availability

The plant material used in the current study was obtained from Dr. AK Joshi and Dr. VK Mishra of Banaras Hindu University, India. The phenotypic datasets created and analysed during the current investigation is available on reasonable request. The source of the genotypic dataset used in this work has been acknowledged in the manuscript and data is available on reasonable request from Dr. AK Joshi and Dr. VK Mishra of Institute of Agricultural Sciences, Banaras Hindu University, India.
