# Supplementary material for: Mapping of QTLs and meta-QTLs for Heterodera avenae Woll. resistance in common wheat (Triticum aestivum L.)
Source: BMC Plant Biol. 2023 Oct 31;23:529. doi: 10.1186/s12870-023-04526-y (PMC10617160; doi:10.1186/s12870-023-04526-y)
Supplement: Supplementary file 2 — Supplementary Material 2 [file 12870_2023_4526_MOESM2_ESM.docx]

**Supplementary Fig. 1**


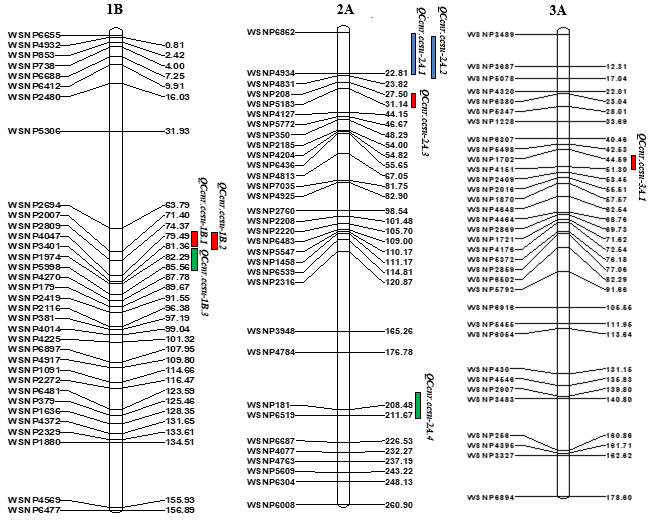


Distribution of QTLs for *H. avenae* resistance on three chromosomes in common wheat detected in the RIL population derived from the cross HUW 468 × C 306 in the present study. Four QTLs detected by CIM alone are indicated by red bar, two detected by ICIM alone are indicated by green bar and the remaining two QTLs detected by both the methods are indicated by blue bar. The names of QTLs and associated markers are shown on the right and the distance (in cM) between markers are shown on the left of each chromosome

**Supplementary Fig. 2**


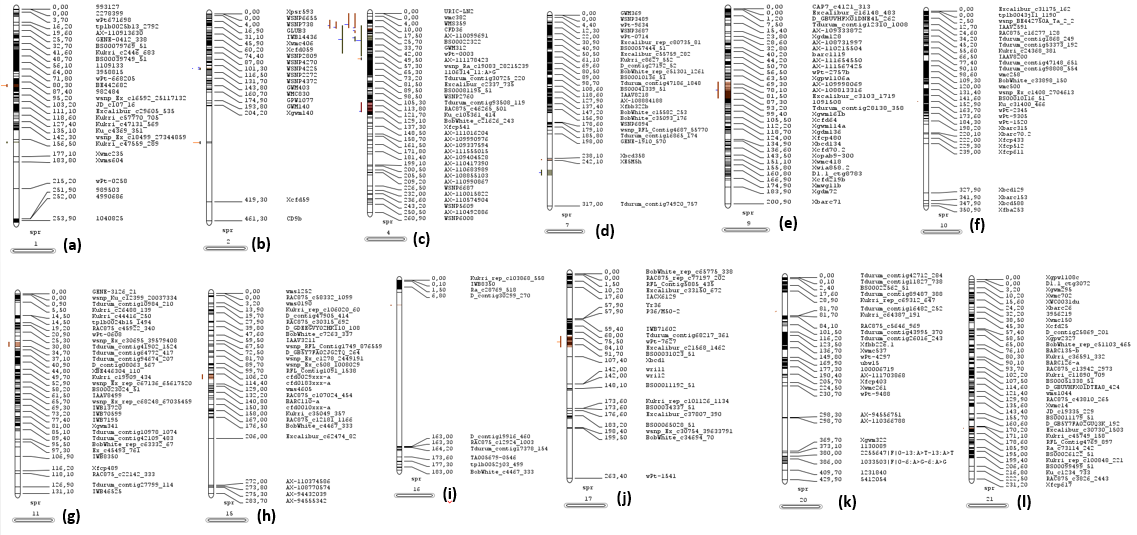


Present figure is showing the projection of QTLs on the consensus map on chromosomes 1A(a), 1B(b), 2A(c), 3A(d), 3D(e), 4A(f), 4B(g), 5D(h), 6A(i), 6B(j), 7B(k) and 7D(l) Bars on left side indicates the projection of QTLs
